# Target Groups for a Short Dexamethasone Course among Critically Ill COVID-19 Patients

**DOI:** 10.1155/2021/5557302

**Published:** 2021-07-16

**Authors:** Armen Oganesyan, Ruslan Menzulin, Yury Surovoy, Andrei Nikiforchin, Kirill Zykov

**Affiliations:** ^1^Clinical Hospital #1 MEDSI, Intensive Care Unit, 1 Pyatnitskoe Shosse 6^th^ km, Otradnoe, Krasnogorsky Rayon, Moscow Oblast 143442, Russia; ^2^Lomonosov Moscow State University, Faculty of Fundamental Medicine, Department of General and Specialized Surgery, 27/1 Lomonosovsky Prospect, Moscow 119192, Russia; ^3^The Institute for Cancer Care, Mercy Medical Center, 227 St. Paul Place, Weinberg Building, Baltimore 21202, MD, USA; ^4^Pulmonology Research Institute, Federal Medical and Biology Agency of Russia, 28 Orekhovyy Bul'var, Moscow 115682, Russia; ^5^Moscow State University of Medicine and Dentistry, 20/1 Delegatskaya Ulitsa, Moscow 127473, Russia

## Abstract

**Introduction:**

Corticosteroids are one of the most promising therapeutic agents for critically ill patients with coronavirus disease 2019 (COVID-19). Despite emerging data, assessed populations and regimens vary, and there are patient subgroups whose response to steroids remains unclear. We aimed to evaluate the outcomes of COVID-19 patients admitted to the intensive care unit (ICU) and treated with a short dexamethasone course to determine which patient categories derive the highest benefit.

**Methods:**

A retrospective cohort study was conducted using a prospectively collected single-center ICU database (April 1–October 1, 2020). Adult COVID-19 patients were assigned to dexamethasone (12 mg × 3 days) and usual care groups. Patient, management, and outcome data were extracted. The primary outcome was the 28-day ICU mortality. Subgroup analysis was performed to assess the impact of dexamethasone on mortality in patients with invasive mechanical ventilation (IMV).

**Results:**

Of 233 patients, 220 (median age: 65 years, 38% female) were included: 83 patients received dexamethasone and 137 received usual care. Overall, 28 (33.7%) and 54 (39.4%) patients in the dexamethasone and usual care groups, respectively, died within 28 days since ICU admission (rate ratio (RR) 0.86; 95% confidence interval (95% CI): 0.59–1.23; *p*=0.405). In the IMV cohort, dexamethasone did not decrease the 28-day mortality compared with usual care (47.5% vs. 62.0%; RR 0.78; 95% CI: 0.57–1.09; *p*=0.107). A subgroup analysis revealed significantly lower 28-day mortality in IMV patients <65 years receiving dexamethasone vs. usual care (22.6% vs. 48.5%; RR 0.47; 95% CI: 0.22–0.98; *p*=0.043), which was not seen in IMV patients ≥65 years (75.0% vs. 71.1%; RR 1.06; 95% CI: 0.79–1.42; *p*=0.719). Patients ≥65 years experienced hyperglycemia, bacterial infection, and septic shock significantly more often than younger patients who received dexamethasone (*p*=0.002, *p*=0.025, and *p* < 0.001, respectively).

**Conclusions:**

A 3-day dexamethasone course is not associated with lower 28-day mortality in critically ill COVID-19 patients, either in the entire ICU cohort or in the IMV. Dexamethasone may significantly reduce the 28-day mortality in IMV patients <65 years, but not in the older IMV subgroup. Dexamethasone administration in patients ≥65 years is associated with a significantly higher rate of adverse events than that in younger patients.

## 1. Introduction

The global coronavirus disease 2019 (COVID-19) pandemic continues despite widespread prevention measures and the recent introduction of effective vaccines [1–7]. Over 300,000 new COVID-19 cases are reported worldwide every day, and up to 18% of patients develop severe disease requiring hospitalization [8, 9]. About 15–35% of hospitalized patients are treated in the intensive care unit (ICU), and up to 91% of them require invasive mechanical ventilation (IMV) [7, 10]. Current data show that the hospital mortality may be up to 20%, and 40% among ICU patients; however, due to insufficient testing for COVID-19, the actual mortality rates might be even higher [7, 11]. Thus, there is an urgent need to identify effective drugs for these patients.

Currently, corticosteroids are one of the most vigorously studied therapeutics showing overall promising results in patients with COVID-19 [12]. The growing interest for these anti-inflammatory drugs is based on their ability to accelerate resolution of acute respiratory distress syndrome (ARDS), which also presents in severe COVID-19 [7, 13–15]. The authors of a retrospective cohort study comprising 201 COVID-19 patients reported that treatment with methylprednisolone was associated with a lower risk of death in patients who developed ARDS [16]. On the contrary, a randomized controlled trial from Brazil including 393 COVID-19 patients did not find a difference in the 28-day mortality between methylprednisolone and placebo [17]. Dexamethasone, a well-known and widely available corticosteroid, was also actively investigated in several studies [18], one of which showed lower 28-day mortality among IMV patients and those on oxygen support than patients who received usual care [11]. However, the experimental arm in this trial received dexamethasone for up to 10 days that could increase the risk of drug-related side effects [19–22]. Thereby, an early 3-day course of methylprednisolone was suggested for use, as it showed improved clinical outcomes in patients with moderate-to-severe COVID-19 [23].

Despite continuously emerging data about the use of steroids in COVID-19, the assessed populations and reported regimens vary significantly, and there are still clinical groups of patients whose response to this therapy remains unclear. Therefore, our study focused on critically ill COVID-19 patients admitted to the ICU and aimed to evaluate their outcomes following treatment with a short 3-day dexamethasone course as well as determine a subgroup of ICU patients who will benefit the most from this approach.

## 2. Materials and Methods

### 2.1. Study Design

We conducted a retrospective cohort study to evaluate the impact of a short 3-day dexamethasone course on outcomes of critically ill COVID-19 patients admitted to the ICU.

### 2.2. Setting

This study was designed and conducted in the ICU of Clinical Hospital #1 MEDSI (Otradnoe, Krasnogorsky Rayon, Moscow Oblast, Russia), a medical center completely reorganized in March 2020 for the management of COVID-19 patients and launched on April 1, 2020. All patients were admitted to the hospital wards via the emergency department or directly to the ICU depending on their condition. The principal indication for ICU admission was respiratory failure defined as peripheral oxygen saturation (SpO_2_) ≤90% with oxygen flow up to 10 L/min and respiratory rate ≥30/min. The usual care in the ICU included, if indicated, oxygen therapy; noninvasive respiratory support; IMV; administration of antibiotics, anticoagulant and vasopressor agents, and enteral and parenteral nutrition; as well as renal-replacement therapy. Some of the patients received hydroxychloroquine or anti-interleukin-6 receptor monoclonal antibodies (i.e., tocilizumab or sarilumab) until they were found ineffective to reduce mortality [24, 25].

### 2.3. Ethics

The Ethical Committee of Clinical Hospital #1 MEDSI determined that this project met the definition of human subject research and approved its conduction (protocol no. 82 dated December 28, 2020).

### 2.4. Data Sources, Participants, and Intervention

A prospectively collected ICU database was reviewed from April 1 to October 1, 2020, and the follow-up continued until December 1, 2020. Male and nonpregnant female patients ≥18 years of age admitted to the ICU were included if they had a laboratory-confirmed (viral RNA detected from nasopharyngeal or oropharyngeal swab samples via reverse transcriptase-polymerase chain reaction (RT-PCR)) or clinically suspected SARS-CoV-2 infection (fever, bilateral ground-glass opacification on chest computed tomography (CT), absence of catarrhal phenomena typical for the influenza virus, negative test for influenza, and disparity of an extent of respiratory distress and percentage of lung injury on chest CT). Patients with another primary diagnosis along with those transferred to another facility and lost for follow-up were excluded. Eligible patients were assigned to two groups based on the administration of a short 3-day dexamethasone course, and their characteristics, management, and outcomes were compared between the groups. The 3-day dexamethasone therapy comprised a 12 mg intravenous dose on day 1 of ICU admission followed by the same dose for 2 more days. The dose and duration of dexamethasone were chosen based on data about its effectiveness in ARDS and the risk of prolonged viral clearance in higher doses of steroids [15, 20, 26]. Although the decision to administer corticosteroids was primarily based on the physician's clinical judgment, working in circumstances of an unstudied disease pandemic and lack of evidence-based approaches certainly influenced these decisions. In our department, steroids were used more routinely at the beginning due to available data on non-COVID-19 ARDS, [14, 15] which is reflected in the 30% rate of dexamethasone administration in April 2020 and even 70% rate in May 2020. Then, we made a pause in using it so routinely and thus only 10% of patients received dexamethasone in June and 25% in July. When the RECOVERY trial was released [11], the rate of steroid administration increased back again in August and September 2020. No patients received dexamethasone or other steroids before the ICU admission.

### 2.5. Assessed Variables

We retrieved the following patient characteristics: age, sex, body mass index (BMI, weight in kilograms divided by the square of height in meters), past and current medical history, vital signs, laboratory test results, percentage of lung tissue injury according to chest CT, and administered medications recorded at the time of ICU admission. Data regarding oxygen therapy (i.e., high-flow nasal cannula and noninvasive respiratory support), IMV, adverse events, and the length of ICU and hospital stay along with ICU readmission were also extracted. Registered bleeding referred to hemorrhage from the gastrointestinal, respiratory, and genitourinary tracts. Occurred thromboembolic events included deep vein thrombosis, splanchnic vein thrombosis, and pulmonary embolism. Hyperglycemia was defined as a random blood glucose level ≥10.0 mmol/L (≥180 mg/dL) [21, 27]. Bacterial infection was identified by present clinical signs of systemic inflammatory response and confirmed by positive bacterial culture. Septic shock was diagnosed according to the 2016 SCCM/ESICM task force criteria in patients with a confirmed bacterial infection requiring vasopressor agents for maintaining mean blood pressure above 65 mm Hg despite fluid resuscitation [28].

### 2.6. Outcomes

The primary outcome was 28-day ICU mortality, which was defined as an all-cause death within 28 days since ICU admission. Secondary outcomes included 28-day mortality in the IMV cohort, extubation or decannulation rate, duration of IMV, lengths of ICU and hospital stay, and the final outcome, which was either discharge from the hospital or death from any cause.

### 2.7. Statistical Analysis

Statistical analysis was conducted using the IBM SPSS Statistics for Windows software (Version 23.0; IBM Corporation, Armonk, NY). Categorical variables are reported as proportions, and continuous variables are presented as medians with interquartile range (IQR). Fisher's exact test and chi-square analysis were used for categorical variables, and the unpaired Mann–Whitney test was used for continuous variables. The primary outcome of 28-day ICU mortality was estimated using the Kaplan–Meier method. By the time of data cut-off on December 1, 2020, the outcome (discharged or deceased) of all patients was known, and discharged patients' data were censored on day 29. The impact of a short dexamethasone course on the 28-day ICU mortality was presented as a rate ratio (RR) with a 95% confidence interval (95% CI). Multivariable logistic regression analysis was performed with 28-day mortality as a dependent variable to evaluate its association with dexamethasone adjusting for other factors. All predictors were included in the analysis, and a stepwise elimination was used to build a final model. The results were presented as an odds ratio (OR) with 95% CI. For all analyses, *p* < 0.05 was considered to indicate statistical significance. Subgroup analysis was performed to assess the impact of dexamethasone on mortality in IMV patients.

## 3. Results

### 3.1. Participants

Of 233 patients in the database, 13 were excluded owing to having non-COVID-19 pneumonia or another primary pathology (urgent surgery, cerebral artery aneurysm rupture, meningitis, etc.); transfer to another facility; and follow-up loss. Finally, 220 patients were included in the study: 83 in the dexamethasone group and 137 in the usual care group.

### 3.2. Patient Characteristics at ICU Admission

The median age was 62 (IQR: 53–70) years in the dexamethasone group and 67 (IQR: 56–75) years in the usual care group (*p*=0.22) (Table 1). Female patients accounted for 31% and 42% in the dexamethasone and usual care groups, respectively (*p*=0.127). BMI ≥ 30 kg/m^2^ was registered in 62% and 57% patients in the dexamethasone and usual care groups, respectively (*p*=0.530). In the dexamethasone group, 70% had positive RT-PCR SARS-CoV-2 vs. 61% in the usual care group (*p*=0.164). Overall, 75% of dexamethasone and 81% of usual care patients had comorbidities (*p*=0.183), as presented in Table 1.

The median respiratory rate was significantly higher (28 (IQR: 24–35) vs. 25 (IQR: 22–30) breaths/min, *p*=0.007), whereas the oxygen saturation of arterial blood was significantly lower (88 (IQR: 80–90) vs. 89 (IQR: 82–94) %, *p*=0.025) in the dexamethasone group than in the usual care group. Fever ≥38°C (≥100.4°F) was recorded in 52% patients in the dexamethasone group versus only 37% patients in the usual care group (*p*=0.026). Other vital signs and quick sequential organ failure assessment (qSOFA) scores are given in Table 1.

The median percentage of lung tissue injury visualized on chest CT was significantly higher in the dexamethasone group than usual care group (64% (IQR: 52–76) vs. 54% (IQR: 36–74)) (*p*=0.017). C-reactive protein (157 (IQR: 97–244) vs. 118 (IQR: 53–209) mg/L, *p*=0.009) and ferritin (691 (IQR: 556–715) vs. 635 (IQR: 376–705) ng/mL, *p*=0.024) levels were significantly higher in the dexamethasone group than the usual care group, respectively. There were no significant intergroup differences in the levels of interleukin-6, D-dimer, and creatinine. There were 31% patients who had procalcitonin ≥0.5 ng/mL in the dexamethasone group versus 32% in the usual care group (*p*=0.916). Arterial blood gas analysis and complete blood counts are in Table 1.

### 3.3. ICU Management

The median start of the studied dexamethasone course was on the 10^th^ (IQR: 8–13) day since the first day of fever along with a median cumulative dose of 36 (IQR: 28–40) mg. The groups were well balanced with respect to other medications and interventions (Table 2). During hospitalization, significantly more patients in the dexamethasone group received sarilumab than in the usual care group (12 (14%) vs. 8 (6%), *p*=0.031). In the ICU, the need for vasopressor agents was higher in the dexamethasone (65%) group than the usual care group (48%) (*p*=0.015). In the dexamethasone group, noninvasive respiratory support was used more frequently (16% vs. 2%) (*p* < 0.001). The intubation and tracheostomy rates were higher in the dexamethasone group than the usual care group (71% vs. 52%, (*p*=0.005) and (78% vs. 56% (*p*=0.009), respectively).

### 3.4. Adverse Events

Bleeding and nonventricular arrhythmia occurred more often in the dexamethasone group than in the usual care group (27% vs. 12%, *p*=0.005, and 31% vs. 19%, *p*=0.037; respectively) (Table 3). On the contrary, the rates of ventricular arrhythmia, *Clostridioides difficile* infection, and thromboembolic events did not differ between the two groups. Hyperglycemia occurred more frequently in the dexamethasone group than in the usual care group (40% vs. 23%, *p*=0.010). The bacterial infection rate was 71% in the dexamethasone group and 61% in the usual care group (*p*=0.141). Septic shock developed in 39 (47%) dexamethasone patients vs. 44 (32%) usual care patients (*p*=0.027*p*).

### 3.5. Primary and Secondary Outcomes

**Figure 1:**
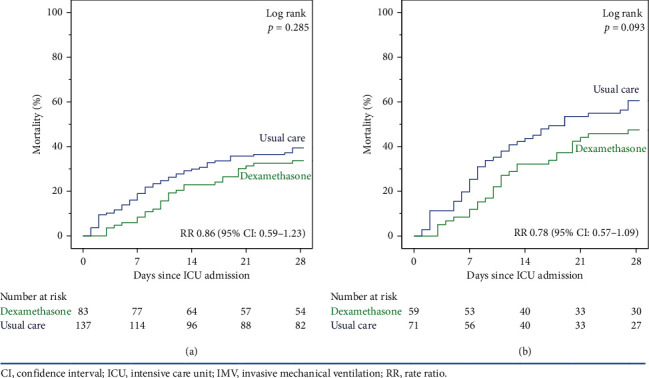
ICU mortality at 28 days. (a) All patients (*n* = 220). (b) IMV patients (*n* = 130).

The 28-day ICU mortality did not differ significantly between the dexamethasone and usual care groups (37.7% vs. 39.4% (RR, 0.86, 95% CI: 0.59–1.23, *p*=0.405) (Figure 1(a)). In IMV patients, there was no significant difference in the 28-day ICU mortality between the two groups: 47.5% vs. 62.0% (RR 0.78; CI 95%: 0.57–1.09, *p*=0.107) (Figure 1(b)). Extubation or decannulation was performed in 50 (38%) IMV patients (Table 2). The rate of extubation or decannulation did not differ significantly between the groups (46% vs. 32%, *p*=0.119). All weaned patients survived and were discharged home without readmission to the ICU. The duration of IMV was significantly longer in the dexamethasone group than the usual care group (16 (IQR: 9–22) vs. 12 (IQR: 5–19) days, *p*=0.026) but did not differ when measured in the survived IMV patients (20 (IQR: 12–31) vs. 17 (IQR: 12–25) days, *p*=0.158).

The initial and total lengths of ICU stay were significantly longer in the dexamethasone group than the usual care group (11 (IQR: 4–21) vs. 5 (IQR: 2–14) days, *p* < 0.001, and 11 (IQR: 4–22) vs. 5 (IQR: 2–15) days, *p*=0.001; respectively) (Table 3). The length of hospital stay did not differ between the two groups (20 (IQR: 12–33) vs. 18 (IQR: 12–26) days, *p*=0.060). The discharge rate did not differ significantly in the dexamethasone and usual care groups (61.4% vs. 56.9%, *p*=0.510).

After the follow-up was completed, the overall ICU mortality was 41.4% and overall mortality in IMV patients was 61.5%.

### 3.6. Subgroup Analysis

**Figure 2:**
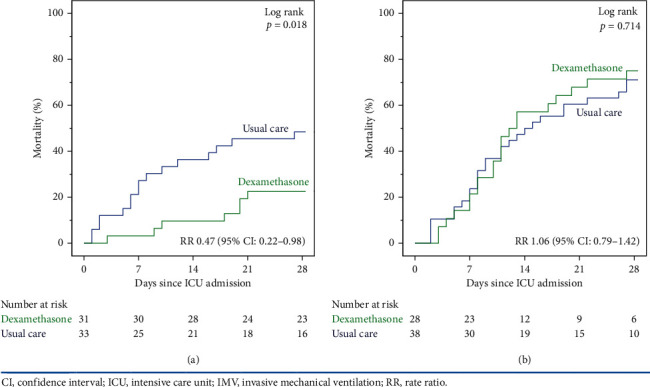
ICU mortality at 28 days in IMV patients. (a) IMV patients <65 years (*n* = 64). (b) IMV patients ≥65 years (*n* = 66).

The 28-day ICU mortality in IMV patients <65 years was significantly lower in the dexamethasone group than the usual care group (22.6% vs. 48.5% (RR: 0.47; 95% CI: 0.22–0.98; *p*=0.043) (Figure 2(a)). On the contrary, this difference was not seen in IMV patients ≥65 years between the dexamethasone and usual care groups (75.0% vs. 71.1% (RR: 1.06, 95% CI: 0.79–1.42, *p*=0.719) (Figure 2(b)). Among patients <65 years who received IMV, the extubation or decannulation rate was significantly higher in the dexamethasone group than the usual care group (68% vs. 42%; *p*=0.042) (Tables S1–S4 in Supplementary Materials).

### 3.7. Multivariable Logistic Regression Analysis

All variables included in the multivariable logistic regression models are presented in Table 4. The final model for all patients included 168 patients and 64 events and dexamethasone use showed to be associated with lower 28-day ICU mortality (OR 0.41; 95% CI: 0.16–1.00, *p*=0.049), whereas age, creatinine level, and the necessity of IMV negatively affected mortality adjusting for other factors. The final model for IMV patients included 104 patients and 55 events, and dexamethasone did not show any association with 28-day ICU mortality. In IMV patients <65 years, there were 52 patients and 17 events in the final model, and dexamethasone administration was associated with lower 28-day ICU mortality (OR 0.17; 95% CI: 0.03–0.84, *p*=0.030) in this subgroup adjusting for other predictors.

## 4. Discussion

Systemic corticosteroids are currently one of the most investigated and discussed medications for COVID-19 management. However, even this approach does not ensure reduced mortality in all COVID-19 patients; therefore, it is important to determine the categories of patients who stand to benefit most from this treatment. The present study was conducted with this particular aim and a focus on critically ill patients with COVID-19 admitted to the ICU.

Data regarding the use of steroids in COVID-19 patients are inconsistent. In a multicenter CoDEX trial comprising 299 ICU patients, the use of dexamethasone did not reduce mortality in COVID-19-induced moderate-to-severe ARDS compared with the control group but significantly increased the number of ventilator-free days for the 28-day period [29]. In the MetCOVID trial, no reduction in 28-day mortality was demonstrated in hospitalized COVID-19 patients treated with methylprednisolone (0.5 mg/kg twice daily for 5 days); however, the authors noted a lower mortality in patients over 60 years [17]. By contrast, in the RECOVERY trial, 6 mg of dexamethasone for 10 days led to lower 28-day mortality in hospitalized COVID-19 patients with the greatest reduction by 36% seen in the most critically ill patients receiving IMV [11]. In the present study, a short 3-day dexamethasone course was not associated with lower 28-day mortality in the entire ICU cohort compared to usual care. However, dexamethasone led to a significant reduction in the 28-day ICU mortality in IMV patients <65 years of age. This result is consistent with data from the RECOVERY trial (benefit of steroids is seen in patients <70 years) and a meta-analysis conducted by the WHO Rapid Evidence Appraisal for COVID-19 Therapies (REACT) Working Group [18].

In our study, we also saw no decrease in mortality in the entire IMV subgroup; however, it does not contradict the RECOVERY findings. We believe it can be explained by the difference in IMV patients' age: 59 years (mean) in the RECOVERY trial vs. 65 years (median) in present study. When we evaluated the effect of dexamethasone therapy solely in a group of IMV patients <65 years with a median age of 55 years, we obtained a significant benefit and even higher (53%) mortality reduction. It should be noted that our study was carried out without limiting the patient inclusion due to strict criteria, which is typical for prospective randomized trials. For instance, in the RECOVERY trial, physicians were able to exclude severely comorbid patients whom they determined to be unsuited candidates for treatment with dexamethasone. As a result, in the group of IMV patients, the number of comorbidities was much lower than in patients who did not require oxygen upon admission. The exclusion of patients with severe comorbid pathology might have contributed to an increase in the steroids' effectiveness in the RECOVERY trial; therefore in this study, we aimed to include as many ICU patients as possible in order to best reflect actual clinical practice.

**Figure 3:**
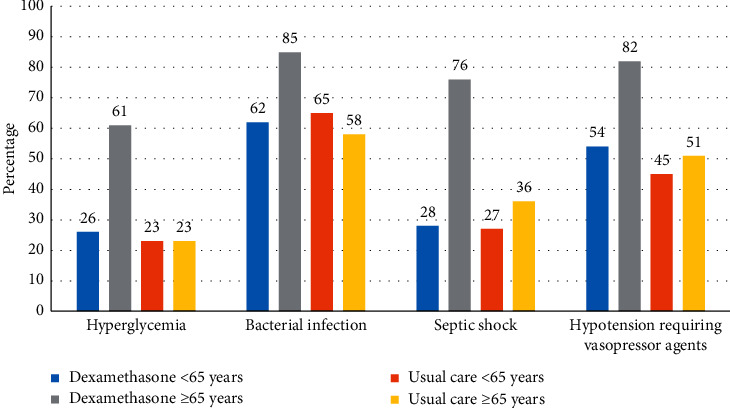
Adverse events in various subgroups of patients.

The most important concern of corticosteroid use in COVID-19 patients is a risk of adverse events, particularly prolonged viral clearance [30, 31] and secondary bacterial and fungal infections [32, 33] previously reported in patients with ARDS of other viral etiology. In our study, patients ≥65 years who received dexamethasone experienced hyperglycemia, bacterial infection, septic shock, and hypotension requiring vasopressor agents significantly more often than younger patients (Figure 3, Tables S5 and S6 in Supplementary Materials). One of the aims was to determine whether these differences were due to either the patients' age and their higher comorbidity rate or the result of dexamethasone use. In this respect, we assessed the outcomes of patients who did not receive dexamethasone (Tables S7 and S8 in Supplementary Material). These complication rates were similar in patients <65 years regardless of steroid use. Moreover, there were no differences in complication rates between the patients <65 and those ≥65 years who did not receive dexamethasone despite more comorbidities in the older cohort. Thus, we suppose that the use of dexamethasone in critically ill ICU patients ≥65 years is associated with an increased number of adverse events that probably explains the absence of a positive impact of dexamethasone on the mortality in this subgroup of COVID-19 patients.

This study has some limitations due to its single-center retrospective design. The decision to use steroids was based primarily on the physician's clinical judgment, which could have led to the use of dexamethasone predominantly in patients with more severe disease. Indeed, variables such as the rate of hypoxia, percentage of lung tissue injury on chest CT, levels of C-reactive protein and ferritin, and the use of noninvasive respiratory support were significantly higher in the dexamethasone group than in the usual care group, which likely explains the higher rates of intubation and IMV in the former group. These confounding factors might have reduced the value of dexamethasone efficacy in patients requiring any level of respiratory support.

## 5. Conclusions

A short 3-day dexamethasone course is not associated with lower 28-day mortality in critically ill COVID-19 patients, either in the entire ICU cohort or in those requiring IMV. However, this therapeutic approach may reduce the 28-day ICU mortality rate in younger IMV patients, but not in the older IMV subgroup. In patients aged ≥65 years, the administration of dexamethasone is associated with a significantly higher rate of adverse events. Further randomized studies are needed to determine the most beneficial regimens of systemic steroid use in elderly patients with severe COVID-19.

## Figures and Tables

**Table 1:** Patient characteristics at ICU admission.

Variable	Dexamethasone (*n* = 83)	Usual care (*n* = 137)	*p* value
Age, years, median (IQR)	62 (53–70)	67 (56–75)	**0.022**
Female sex, *n* (%)	26 (31)	57 (42)	0.127
BMI ≥30, kg/m^2^, *n* (%)	40/65 (62)	52/92 (57)	0.530
Positive RT-PCR SARS-CoV-2, *n* (%)	58 (70)	83 (61)	0.164
Comorbidities, *n* (%)	62 (75)	111 (81)	0.183
Hypertension	45 (54)	88 (64)	0.060
Other CVD	22 (27)	46 (34)	0.194
Chronic lung disease	10 (12)	16 (12)	0.981
Chronic kidney disease	2 (2)	11 (8)	0.075
Diabetes mellitus	19 (23)	41 (30)	0.188
Malignancy	10 (12)	15 (11)	0.869
Systolic BP, mm Hg, median (IQR)	130 (120–140)	130 (117–140)	0.708
Diastolic BP, mm Hg, median (IQR)	75 (70–85)	75 (69–80)	0.625
HR, beats/min, median (IQR)	84 (75–91)	85 (74–100)	0.585
RR, breaths/min, median (IQR)	28 (24–35)	25 (22–30)	**0.007**
SaO_2_, %, median (IQR)	88 (80–90)	89 (82–94)	**0.025**
Fever ≥38^o^ C (≥100.4°F), *n* (%)	43 (52)	51 (37)	**0.026**
qSOFA, *n* (%)			
0	3 (4)	16 (12)	**0.039**
1	60 (72)	73 (53)	**0.005**
2	13 (16)	29 (21)	0.314
3	7 (8)	19 (14)	0.226
Lung injury on chest CT scan, %, median (IQR)	64 (52–76)	54 (36–74)	**0.017**
CRP, mg/L, median (IQR)	157 (97–244)	118 (53–209)	**0.009**
Ferritin, ng/mL, median (IQR)	691 (556–715)	635 (376–705)	**0.024**
Interleukin-6, pg/mL, median (IQR)	92 (35–156)	81 (24–212)	0.892
D-dimer, ng/mL, median (IQR)	1,424 (665–3,446)	1,556 (810–3,912)	0.575
Creatinine, mcmol/L, median (IQR)	92 (75–117)	91 (71–118)	0.850
Procalcitonin ≥0.5 ng/mL, *n* (%)	23/74 (31)	35/110 (32)	0.916
Arterial blood gas analysis, median (IQR)			
Blood pH	7.41 (7.33–7.46)	7.38 (7.32–7.43)	0.150
PaO_2_, mm Hg	83 (68–110)	89 (68–113)	0.812
PaCO_2_, mm Hg	39 (33–48)	38 (32–51)	0.983
Complete blood count, median (IQR)			
Hemoglobin, g/L	126 (113–137)	124 (103–139)	0.785
Leukocytes, ×10^9^/L	8.0 (6.0–11.5)	8.2 (5.2–12.0)	0.948
Neutrophils, ×10^9^/L	6.6 (4.6–10.0)	6.8 (3.9–10.4)	0.836
Lymphocytes, ×10^9^/L	0.7 (0.4–1.0)	0.7 (0.5–1.1)	0.579
Platelets, ×10^9^/L	233 (169–318)	200 (146–283)	0.134

BMI, body mass index; BP, blood pressure; CRP, C-reactive protein; CT, computed tomography; CVD, cardiovascular disease; HR, heart rate; ICU, intensive care unit; IQR, interquartile range; PaCO_2_, partial pressure of carbon dioxide; PaO_2_, partial pressure of oxygen; qSOFA, quick sequential organ failure assessment score; RR, respiratory rate; RT-PCR, reverse transcription polymerase chain reaction; SaO_2_, oxygen saturation of arterial blood; SARS-CoV-2, severe acute respiratory syndrome coronavirus 2. Bold values denote statistical significance.

**Table 2:** ICU management.

Variable	Dexamethasone (*n* = 83)	Usual care (*n* = 137)	*p* value
Dexamethasone therapy			
Therapy start since the 1^st^ day of fever, days, median (IQR)	10 (8–13)	NA	NA
Duration of therapy, days, median (IQR)	3 (3–3)		
Cumulative dose, mg, median (IQR)	36 (28–40)		
Hydroxychloroquine, *n* (%)	19 (23)	30 (22)	0.864
Sarilumab, *n* (%)	12 (14)	8 (6)	**0.031**
Tocilizumab, *n* (%)	14 (17)	13 (9)	0.106
Antibiotics, *n* (%)	81 (98)	133 (97)	0.822
DVT prophylaxis, *n* (%)	83 (100)	132 (96)	0.078
RBC transfusion, *n* (%)	21 (25)	27 (20)	0.330
Dialysis, *n* (%)	24 (29)	27 (20)	0.117
Vasopressor agents, *n* (%)	54 (65)	66 (48)	**0.015**
NEpi, *n* (%)	34 (41)	41 (30)	0.469
NEpi + Epi, *n* (%)	18 (22)	22 (16)	
NEpi + Epi + other agents, *n* (%)	2 (2)	3 (2)	
Highest dose, mcg/kg, median (IQR)	0.7 (0.2–1.2)	0.6 (0.2–1.0)	0.339
Nutrition, *n* (%)			
Oral intake	20 (24)	60 (44)	**0.003**
NG feeding tube	50 (60)	71 (52)	0.224
NG feeding tube + PN	13 (16)	6 (4)	**0.004**
O_2_ therapy, *n* (%)			
Nasal cannula	54 (65)	102 (74)	0.137
HFNC	14 (17)	25 (18)	0.867
Noninvasive mechanical ventilation	13 (16)	3 (2)	**<0.001**
Intubation, *n* (%)	59 (71)	71 (52)	**0.005**
Tracheostomy, *n* (%)	46/59 (78)	40/71 (56)	**0.009**
Day of tracheostomy after intubation, days, median (IQR)	5 (3–7)	6 (3–14)	0.563
ECMO, *n* (%)	1 (1.2)	2 (1.5)	0.865
Extubation/decannulation, *n* (%)	27/59 (46)	23/71 (32)	0.119
Duration of ventilation, days, median (IQR)	16 (9–22)	12 (5–19)	**0.026**
Duration of ventilation in survivors, days, median (IQR)	20 (12–31)	17 (12–25)	0.158

CPAP, continuous positive airway pressure; DVT, deep vein thrombosis; ECMO, extracorporeal membrane oxygenation; Epi, epinephrine; HFNC, high-flow nasal cannula; ICU, intensive care unit; IQR, interquartile range; NA, not applicable; NEpi, norepinephrine; NG, nasogastric; PN, parenteral nutrition; RBC, red blood cells. Bold values denote statistical significance.

**Table 3:** Adverse events and outcomes.

Variable	Dexamethasone (*n* = 83)	Usual care (*n* = 137)	*p* value
Bleeding, *n* (%)	22 (27)	16 (12)	**0.005**
Nonventricular arrhythmia, *n* (%)	26 (31)	26 (19)	**0.037**
Ventricular arrhythmia, *n* (%)	1 (1)	4 (3)	0.408
*Clostridioides difficile* infection, *n* (%)	5 (6)	5 (4)	0.412
Thromboembolic events, *n* (%)	17 (20)	24 (18)	0.584
Hyperglycemia, *n* (%)	33 (40)	32 (23)	**0.010**
Bacterial infection, *n* (%)	59 (71)	84 (61)	0.141
Septic shock, *n* (%)	39 (47)	44 (32)	**0.027**
Initial ICU stay, days, median (IQR)	11 (4–21)	5 (2–14)	**<0.001**
ICU readmission, *n* (%)	5 (6)	12 (9)	0.476
Total ICU stay, days, median (IQR)	11 (4–22)	5 (2–15)	**0.001**
Length of hospital stay, days, median (IQR)	20 (12–33)	18 (12–26)	0.060
28-day ICU mortality, *n* (%)	28 (33.7)	54 (39.4)	0.398
Outcome, *n* (%)			
Discharged	51 (61.4)	78 (56.9)	0.510
Deceased	32 (38.6)	59 (43.1)	

ICU, intensive care unit; IQR, interquartile range; NA, not applicable. Bold values denote statistical significance.

**Table 4:** Multivariable stepwise logistic regression analysis of 28-day ICU mortality predictors.

Variable	All patients			IMV patients			IMV patients <65 years		
	OR	95% CI	*p* value	OR	95% CI	*p* value	OR	95% CI	*p* value
Dexamethasone	**0.41**	**0.16–1.00**	**0.049**	—	—	—	**0.17**	**0.03–0.84**	**0.030**
Age	1.12	1.07–1.16	**<0.001**	1.11	1.06–1.17	**<0.001**	1.24	1.05–1.47	**0.013**
Male sex	—	—	—	—	—	—	—	—	—
Comorbidities	—	—	—	—	—	—	—	—	—
Lactate	—	—	—	—	—	—	—	—	—
Creatinine	1.01	1.00–1.01	**0.036**	1.02	1.00–1.03	**0.014**	1.03	1.00–1.05	**0.022**
Lung injury on chest CT scan	—	—	—	—	—	—	—	—	—
Anti-interleukin-6 receptor MABs	—	—	—	–—	—	—	—	—	—
IMV	24.60	7.35–82.80	**<0.001**	NA	NA	NA	NA	NA	NA

CT, computed tomography; ICU, intensive care unit; IMV, invasive mechanical ventilation; MABs, monoclonal antibodies; NA, not applicable. Bold values denote statistical significance.

## Data Availability

The data sets used and analyzed during the current study are available from the corresponding author on reasonable request. The participant data were de-identified and are kept confidential.

## Abbreviations

ARDS: Acute respiratory distress syndrome
BMI: Body mass index
CI: Confidence interval
COVID-19: Coronavirus disease 2019
CT: Computed tomography
ESICM: European Society of Intensive Care Medicine
ICU: Intensive care unit
IMV: Invasive mechanical ventilation
IQR: Interquartile range
REACT: Rapid Evidence Appraisal for COVID-19 Therapies
RNA: Ribonucleic acid
RR: Rate ratio
RT-PCR: Reverse transcriptase-polymerase chain reaction
SARS-CoV-2: Severe acute respiratory syndrome coronavirus 2
SCCM: Society of Critical Care Medicine
SpO_2_: Peripheral oxygen saturation
qSOFA score: Quick sequential organ failure assessment score
WHO: World Health Organization.

## Supplementary Materials

Supplementary Materials Table S1: characteristics of IMV patients <65 years at ICU admission. Table S2: management and outcomes of IMV patients <65 years. Table S3: characteristics of IMV patients ≥65 years at ICU admission. Table S4: management and outcomes of IMV patients ≥65 years. Table S5: characteristics of a dexamethasone cohort at ICU admission. Table S6: management and outcomes of a dexamethasone cohort. Table S7: characteristics of a usual care cohort at ICU admission. Table S8: management and outcomes of a usual care cohort.
