# Supplementary material for: Target Groups for a Short Dexamethasone Course among Critically Ill COVID-19 Patients
Source: Crit Care Res Pract. 2021 Jul 16;2021:5557302. doi: 10.1155/2021/5557302 (PMC8645403; doi:10.1155/2021/5557302)
Supplement: Supplementary Materials — Table S1: characteristics of IMV patients <65 years at ICU admission. Table S2: management and outcomes of IMV patients <65 years. Table S3: characteristics of IMV patients ≥65 years at ICU admission. Table S4: management and outcomes of IMV patients ≥65 years. Table S5: characteristics of a dexamethasone cohort at ICU admission. Table S6: management and outcomes of a dexamethasone cohort. Table S7: characteristics of a usual care cohort at ICU admission. Table S8: management and outcomes of a usual care cohort. [file 5557302.f1.docx]

**SUPPLEMENTARY MATERIALS**

This document has been provided by the authors to give readers additional information about their study.

Supplement to: Target Groups for a Short Dexamethasone Course among Critically Ill COVID-19 Patients.

(Document last updated on July 7, 2021)

**CONTENTS**

**TABLE S1.** Characteristics of IMV patients <65 years at ICU admission 3

**TABLE S2.** Management and outcomes of IMV patients <65 years 4

**TABLE S3.** Characteristics of IMV patients ≥65 years at ICU admission 5

**TABLE S4.** Management and outcomes of IMV patients ≥65 years 6

**TABLE S5.** Characteristics of a dexamethasone cohort at ICU admission 7

**TABLE S6.** Management and outcomes of a dexamethasone cohort 8

**TABLE S7.** Characteristics of a usual care cohort at ICU admission 9

**TABLE S8.** Management and outcomes of a usual care cohort 10

**TABLE S1.** Characteristics of IMV patients <65 years at ICU admission

| **Variable** | **Dexamethasone**  (n=31) | **Usual Care**  (n=33) | ***p* value** |  |
| --- | --- | --- | --- | --- |
| Age, years, median (IQR) | 55 (49-59) | 54 (49-60) | 0.819 |  |
| Female sex, n (%) | 13 (42) | 10 (30) | 0.332 |  |
| BMI ≥30, kg/m2, n (%) | 22 / 28 (79) | 20 / 25 (80) | 0.898 |  |
| Positive RT-PCR SARS-CoV-2, n (%) | 22 (71) | 19 (58) | 0.264 |  |
| Comorbidities, n (%) | 20 (65) | 25 (76) | 0.325 |  |
| Hypertension | 13 (42) | 17 (55) | 0.309 |  |
| Other CVD | 3 (10) | 7 (23) | 0.167 |  |
| Chronic lung disease | 2 (7) | 5 (16) | 0.425 |  |
| Chronic kidney disease | 1 (3) | 1 (3) | 1.000 |  |
| Diabetes mellitus | 7 (23) | 9 (29) | 0.562 |  |
| Malignancy | 3 (10) | 3 (10) | 1.000 |  |
| Systolic BP, mm Hg, median (IQR) | 130 (118-140) | 131 (120-150) | 0.291 |  |
| Diastolic BP, mm Hg, median (IQR) | 70 (70-80) | 75 (69-80) | 0.835 |  |
| HR, beats/min, median (IQR) | 84 (76-92) | 95 (80-110) | 0.057 |  |
| RR, breaths/min, median (IQR) | 30 (25-36) | 32 (25-35) | 0.814 |  |
| SaO_2_, %, median (IQR) | 85 (77-89) | 84 (78-90) | 0.783 |  |
| Fever ≥38^o^ C (≥100.4^o^ F), n (%) | 23 (77) | 22 (73) | 0.766 |  |
| qSOFA, n (%) |  |  |  |  |
| 0 | 0 (0) | 1 (3) | 1.000 |  |
| 1 | 21 (68) | 19 (58) | 0.401 |  |
| 2 | 4 (13) | 6 (18) | 0.734 |  |
| 3 | 6 (19) | 7 (21) | 0.854 |  |
| Lung injury on chest CT scan, %, median (IQR) | 73 (61-78) | 61 (43-73) | **0.022** |  |
| CRP, mg/L, median (IQR) | 171 (109-238) | 171 (73-222) | 0.938 |  |
| Ferritin, ng/mL, median (IQR) | 692 (619-725) | 692 (612-716) | 0.796 |  |
| Interleukin-6, pg/mL, median (IQR) | 88 (61-165) | 73 (22-292) | 0.981 |  |
| Procalcitonin ≥0.5 ng/mL, n (%) | 7 / 28 (25) | 11 / 27 (41) | 0.214 |  |
| d-dimer, ng/mL, median (IQR) | 1,503 (665-3,717) | 2,011 (630-4,127) | 0.767 |  |
| Creatinine, mcmol/L, median (IQR) | 88 (75-110) | 86 (75-109) | 0.731 |  |
| Arterial blood gas analysis, median (IQR) |  |  |  |  |
| Blood pH | 7.42 (7.34-7.46) | 7.37 (7.20-7.42) | 0.114 |  |
| PaO_2_, mm Hg | 92 (67-102) | 83 (58-99) | 0.252 |  |
| PaCO_2_, mm Hg | 41 (36-47) | 43 (33-60) | 0.486 |  |
| Complete blood count, median (IQR) |  |  |  |  |
| Hemoglobin, g/L | 119 (113-133) | 131 (118-146) | 0.057 |  |
| Leukocytes x10^9^/L | 8.0 (6.0-11.9) | 9.9 (5.9-14.0) | 0.554 |  |
| Neutrophils x10^9^/L | 6.6 (4.8-10.3) | 8.3 (3.7-12.2) | 0.643 |  |
| Lymphocytes x10^9^/L | 0.7 (0.5-1.2) | 0.6 (0.4-1.1) | 0.492 |  |
| Platelets x10^9^/L | 225 (175-289) | 200 (163-270) | 0.460 |  |
| BMI, body mass index; BP, blood pressure; CRP, C-reactive protein; CT, computed tomography; CVD, cardiovascular disease; HR, heart rate; ICU, intensive care unit; IQR, interquartile range; PaCO2, partial pressure of carbon dioxide; PaO2, partial pressure of oxygen; qSOFA, quick sequential organ failure assessment score; RR, respiratory rate; RT-PCR, reverse transcription polymerase chain reaction; SaO2, oxygen saturation of arterial blood; SARS-CoV-2, severe acute respiratory syndrome coronavirus 2 | | | | |

**TABLE S2.** Management and outcomes of IMV patients <65 years

| **Variable** | | | **Dexamethasone**  (n=31) | **Usual Care**  (n=33) | ***p* value** | |
| --- | --- | --- | --- | --- | --- | --- |
| Dexamethasone therapy | |  |  |  |  | |
| Therapy start*****, days, median (IQR) | | | 10 (7-13) | NA | NA | |
| Duration of therapy, days, median (IQR) | | | 3 (3-3) |  |  |  |
| Cumulative dose, mg, median (IQR) | | | 36 (36-40) |  |  |  |
| Hydroxychloroquine, n (%) | | | 10 (32) | 11 (33) | 0.927 | |
| Sarilumab, n (%) | | | 4 (13) | 1 (3) | 0.190 | |
| Tocilizumab, n (%) | | | 9 (29) | 6 (18) | 0.306 | |
| Antibiotics, n (%) | | | 31 (100) | 33 (100) | NA | |
| DVT prophylaxis, n (%) | | | 31 (100) | 32 (97) | 1.000 | |
| RBC transfusion, n (%) | | 11 (36) | 10 (30) | 0.659 | |  |
| Dialysis, n (%) | | 13 (42) | 10 (30) | 0.332 | |  |
| Vasopressor agents, n (%) | | | 26 (84) | 26 (79) | 0.603 | |
| NEpi only, n (%) | | | 19 (61) | 17 (52) | 0.820 | |
| NEpi + Epi, n (%) | | | 6 (19) | 8 (24) |  |  |
| NEpi + Epi + other agents, n (%) | | | 1 (3) | 1 (3) |  |  |
| Highest dose, mcg/kg, median (IQR) | | | 0.4 (0.2-1.2) | 0.8 (0.2-1.0) | 0.962 | |
| Nutrition, n (%) | | |  |  |  | |
| Oral intake | | | 0 (0) | 4 (12) | 0.114 | |
| NG feeding tube | | | 20 (65) | 29 (88) | **0.027** | |
| NG feeding tube + PN | | | 11 (35) | 0 (0) | **<0.001** | |
| O_2_ therapy, n (%) | |  |  |  | |  |
| Nasal cannula | | 19 (61) | 20 (61) | 0.955 | |  |
| HFNC | | 5 (16) | 8 (24) | 0.420 | |  |
| Non-invasive mechanical ventilation | | 7 (23) | 2 (6) | 0.078 | |  |
| Tracheostomy, n (%) | | 30 (97) | 23 (70) | **0.004** | |  |
| Day of tracheostomy after intubation, n (%) | | 6 (4-7) | 5 (3-10) | 0.766 | |  |
| ECMO, n (%) | | | 1 (3) | 2 (6) | 1.000 | |
| Extubation / Decannulation, n (%) | | 21 (68) | 14 (42) | **0.042** | |  |
| Duration of ventilation, days, median (IQR) | | 20 (12-33) | 12 (5-17) | **0.001** | |  |
| Bleeding, n (%) | | 13 (42) | 9 (27) | 0.217 | |  |
| Non-ventricular arrhythmia, n (%) | | 7 (23) | 8 (24) | 0.875 | |  |
| Ventricular arrhythmia, n (%) | | 1 (3) | 0 (0) | 0.484 | |  |
| *Clostridioides difficile* infection, n (%) | | 3 (10) | 0 (0) | 0.108 | |  |
| Thromboembolic events, n (%) | | 12 (39) | 9 (27) | 0.330 | |  |
| Hyperglycemia, n (%) | | 11 (36) | 11 (33) | 0.856 | |  |
| Bacterial infection, n (%) | | 30 (97) | 33 (100) | 0.484 | |  |
| Septic shock, n (%) | | 14 (45) | 16 (49) | 0.790 | |  |
| Initial ICU stay, days, median (IQR) | | 21 (12-35) | 15 (7-19) | **0.007** | |  |
| ICU readmission, n (%) | | 1 (3) | 1 (3) | 1.000 | |  |
| Total ICU stay, days, median (IQR) | | 21 (13-35) | 15 (7-19) | **0.007** | |  |
| Length of hospital stay, days, median (IQR) | | 31 (20-46) | 20 (10-27) | **0.002** | |  |
| 28-day ICU mortality, n (%) | | 7 (22.6) | 16 (48.5) | **0.031** | |  |
| Outcome, n (%) | |  |  |  | |  |
| Discharged | | 21 (67.7) | 14 (42.4) | **0.042** | |  |
| Deceased | | 10 (32.3) | 19 (57.6) |  |  |  |
| DVT, deep vein thrombosis; ECMO, extracorporeal membrane oxygenation; Epi, epinephrine; HFNC, high-flow nasal cannula; ICU, intensive care unit; IQR, interquartile range; NA, not applicable; NEpi, norepinephrine; NG, nasogastric; PN, parenteral nutrition; RBC, red blood cells  *** since the first day of fever** | | | | | |  |

**TABLE S3.** Characteristics of IMV patients ≥65 years at ICU admission

| **Variable** | **Dexamethasone**  (n=28) | **Usual Care**  (n=38) | ***p* value** |  |
| --- | --- | --- | --- | --- |
| Age, years, median (IQR) | 75 (69-80) | 73 (70-81) | 0.871 |  |
| Female sex, n (%) | 10 (36) | 17 (45) | 0.461 |  |
| BMI ≥30, kg/m2, n (%) | 10 / 21 (48) | 13 / 26 (50) | 0.871 |  |
| Positive RT-PCR SARS-CoV-2, n (%) | 22 (79) | 29 (76) | 0.829 |  |
| Comorbidities, n (%) | 25 (89) | 36 (95) | 0.307 |  |
| Hypertension | 20 (71) | 28 (74) | 0.561 |  |
| Other CVD | 14 (50) | 16 (42) | 0.735 |  |
| Chronic lung disease | 4 (14) | 4 (11) | 0.721 |  |
| Chronic kidney disease | 1 (4) | 8 (21) | 0.066 |  |
| Diabetes mellitus | 8 (29) | 14 (37) | 0.389 |  |
| Malignancy | 7 (25) | 6 (16) | 0.381 |  |
| Systolic BP, mm Hg, median (IQR) | 133 (122-140) | 125 (117-140) | 0.426 |  |
| Diastolic BP, mm Hg, median (IQR) | 75 (68-83) | 75 (65-85) | 0.907 |  |
| HR, beats/min, median (IQR) | 83 (73-99) | 82 (74-102) | 1.000 |  |
| RR, breaths/min, median (IQR) | 30 (25-40) | 25 (22-31) | **0.021** |  |
| SaO_2_, %, median (IQR) | 86 (81-90) | 86 (80-91) | 0.640 |  |
| Fever ≥38^o^ C (≥100.4^o^ F), n (%) | 16 (57) | 18 (47) | 0.469 |  |
| qSOFA, n (%) |  |  |  |  |
| 0 | 0 (0) | 1 (3) | 1.000 |  |
| 1 | 21 (75) | 20 (53) | 0.064 |  |
| 2 | 6 (21) | 9 (24) | 0.829 |  |
| 3 | 1 (4) | 8 (21) | 0.067 |  |
| Lung injury on chest CT scan, %, median (IQR) | 64 (55-76) | 63 (47-81) | 0.674 |  |
| CRP, mg/L, median (IQR) | 215 (97-271) | 156 (92-222) | 0.158 |  |
| Ferritin, ng/mL, median (IQR) | 694 (485-734) | 631 (448-705) | 0.274 |  |
| Interleukin-6, pg/mL, median (IQR) | 109 (27-264) | 151 (83-473) | 0.243 |  |
| Procalcitonin ≥0.5 ng/mL, n (%) | 11 / 26 (42) | 12 / 30 (40) | 0.861 |  |
| Creatinine, mcmol/L, median (IQR) | 102 (76-124) | 103 (71-130) | 0.914 |  |
| d-dimer, ng/mL, median (IQR) | 2,287 (1,076-5,566) | 1,912 (1,227-6,107) | 0.926 |  |
| Arterial blood gas analysis, median (IQR) |  |  |  |  |
| Blood pH | 7.37 (7.20-7.45) | 7.39 (7.31-7.43) | 0.630 |  |
| PaO_2_, mm Hg | 86 (77-116) | 91 (75-113) | 0.950 |  |
| PaCO_2_, mm Hg | 43 (29-55) | 38 (33-51) | 0.937 |  |
| Complete blood count, median (IQR) |  |  |  |  |
| Hemoglobin, g/L | 122 (107-136) | 114 (88-125) | 0.105 |  |
| Leukocytes x10^9^/L | 9.0 (6.0-13.9) | 9.0 (6.0-15.0) | 0.736 |  |
| Neutrophils x10^9^/L | 7.8 (4.9-11.0) | 7.9 (5.8-12.9) | 0.483 |  |
| Lymphocytes x10^9^/L | 0.7 (0.4-0.9) | 0.6 (0.4-0.7) | 0.915 |  |
| Platelets x10^9^/L | 226 (171-325) | 209 (146-271) | 0.281 |  |
| BMI, body mass index; BP, blood pressure; CRP, C-reactive protein; CT, computed tomography; CVD, cardiovascular disease; HR, heart rate; ICU, intensive care unit; IQR, interquartile range; PaCO2, partial pressure of carbon dioxide; PaO2, partial pressure of oxygen; qSOFA, quick sequential organ failure assessment score; RR, respiratory rate; RT-PCR, reverse transcription polymerase chain reaction; SaO2, oxygen saturation of arterial blood; SARS-CoV-2, severe acute respiratory syndrome coronavirus 2 | | | | |

**TABLE S4.** Management and outcomes of IMV patients ≥65 years

| **Variable** | | | **Dexamethasone**  (n=28) | | **Usual Care**  (n=38) | | ***p* value** | | |
| --- | --- | --- | --- | --- | --- | --- | --- | --- | --- |
| Dexamethasone therapy | |  |  | |  | |  | | |
| Therapy start*****, days, median (IQR) | | | 11 (8-15) | | NA | | NA | | |
| Duration of therapy, days, median (IQR) | | | 3 (3-4) | |  | |  | | |
| Cumulative dose, mg, median (IQR) | | | 36 (36-48) | |  | |  | | |
| Hydroxychloroquine, n (%) | | | 4 (14) | | 4 (11) | | 0.714 | | |
| Sarilumab, n (%) | | | 4 (14) | | 4 (11) | | 0.714 | | |
| Tocilizumab, n (%) | | | 4 (14) | | 2 (5) | | 0.389 | | |
| Antibiotics, n (%) | | | 28 (100) | | 38 (100) | | NA | | |
| DVT prophylaxis, n (%) | | | 28 (100) | | 38 (100) | | NA | | |
| RBC transfusion, n (%) | | 10 (36) | | 13 (34) | | 0.899 | | |  |
| Dialysis, n (%) | | 11 (39) | | 14 (37) | | 0.840 | | |  |
| Vasopressor agents, n (%) | | | 27 (96) | | 34 (90) | | 0.385 | | |
| NEpi only, n (%) | | | 14 (50) | | 18 (47) | | 0.404 | | |
| NEpi + Epi, n (%) | | | 12 (43) | | 14 (37) | |  |  |  |
| NEpi + Epi + other agents, n (%) | | | 1 (4) | | 2 (4) | |  |  |  |
| Highest dose, mcg/kg, median (IQR) | | | 0.8 (0.6-1.0) | | 0.6 (0.3-1.0) | | 0.259 | | |
| Nutrition, n (%) | | |  | |  | |  | | |
| Oral intake | | | 0 (0) | | 1 (3) | | 1.000 | | |
| NG feeding tube | | | 26 (93) | | 32 (84) | | 0.451 | | |
| NG feeding tube + PN | | | 2 (7) | | 5 (13) | | 0.689 | | |
| O_2_ therapy, n (%) | |  | |  | |  | | |  |
| Nasal cannula | | 17 (61) | | 23 (61) | | 0.988 | | |  |
| HFNC | | 4 (14) | | 11 (29) | | 0.160 | | |  |
| Non-invasive mechanical ventilation | | 8 (29) | | 2 (5) | | **0.014** | | |  |
| Tracheostomy, n (%) | | 16 (57) | | 17 (45) | | 0.319 | | |  |
| Day of tracheostomy after intubation, n (%) | | 5 (3-7) | | 7 (3-19) | | 0.205 | | |  |
| ECMO, n (%) | | | 0 (0) | | 0 (0) | | NA | | |
| Extubation / Decannulation, n (%) | | 6 (21) | | 9 (24) | | 0.829 | | |  |
| Duration of ventilation, days, median (IQR) | | 11 (8-18) | | 13 (4-21) | | 0.948 | | |  |
| Bleeding, n (%) | | 9 (32) | | 7 (18) | | 0.199 | | |  |
| Non-ventricular arrhythmia, n (%) | | 18 (64) | | 13 (34) | | **0.016** | | |  |
| Ventricular arrhythmia, n (%) | | 0 (0) | | 2 (5) | | 0.504 | | |  |
| *Clostridioides difficile* infection, n (%) | | 2 (7) | | 3 (8) | | 1.000 | | |  |
| Thromboembolic events, n (%) | | 5 (18) | | 11 (29) | | 0.299 | | |  |
| Hyperglycemia, n (%) | | 19 (68) | | 15 (40) | | **0.023** | | |  |
| Bacterial infection, n (%) | | 28 (100) | | 37 (97) | | 1.000 | | |  |
| Septic shock, n (%) | | 25 (89) | | 26 (68) | | **0.046** | | |  |
| Initial ICU stay, days, median (IQR) | | 12 (8-19) | | 13 (7-26) | | 1.000 | | |  |
| ICU readmission, n (%) | | 3 (11) | | 2 (5) | | 0.643 | | |  |
| Total ICU stay, days, median (IQR) | | 12 (8-21) | | 14 (7-26) | | 0.897 | | |  |
| Length of hospital stay, days, median (IQR) | | 14 (11-32) | | 19 (8-29) | | 0.969 | | |  |
| 28-day ICU mortality, n (%) | | 21 (75.0) | | 27 (71.1) | | 0.722 | | |  |
| Outcome, n (%) | |  | |  | |  | | |  |
| Discharged | | 6 (21.4) | | 9 (23.7) | | 0.829 | | |  |
| Deceased | | 22 (78.6) | | 29 (76.3) | |  |  |  |  |
| DVT, deep vein thrombosis; ECMO, extracorporeal membrane oxygenation; Epi, epinephrine; HFNC, high-flow nasal cannula; ICU, intensive care unit; IQR, interquartile range; NA, not applicable; NEpi, norepinephrine; NG, nasogastric; PN, parenteral nutrition; RBC, red blood cells  *** since the first day of fever** | | | | | | | | |  |

**TABLE S5.** Characteristics of a dexamethasone cohort at ICU admission

| **Variable** | **<65 years**  (n=50) | **≥65 years**  (n=33) | ***p* value** |  |
| --- | --- | --- | --- | --- |
| Age, years, median (IQR) | 56 (48-60) | 73 (68-79) | **<0.001** |  |
| Female sex, n (%) | 16 (32) | 10 (30) | 0.870 |  |
| BMI ≥30, kg/m2, n (%) | 29 / 40 (73) | 11 / 25 (44) | **0.022** |  |
| Positive RT-PCR SARS-CoV-2, n (%) | 33 (66) | 25 (76) | 0.343 |  |
| Comorbidities, n (%) | 32 (64) | 30 (91) | **0.006** |  |
| Hypertension | 22 (44) | 23 (70) | **0.027** |  |
| Other CVD | 8 (16) | 14 (42) | **0.009** |  |
| Chronic lung disease | 4 (8) | 6 (18) | 0.191 |  |
| Chronic kidney disease | 1 (2) | 1 (3) | 1.000 |  |
| Diabetes mellitus | 10 (20) | 9 (27) | 0.470 |  |
| Malignancy | 3 (6) | 7 (21) | 0.081 |  |
| Systolic BP, mm Hg, median (IQR) | 130 (119-140) | 130 (122-140) | 0.378 |  |
| Diastolic BP, mm Hg, median (IQR) | 75 (70-85) | 75 (70-80) | 0.862 |  |
| HR, beats/min, median (IQR) | 84 (75-90) | 83 (73-99) | 0.926 |  |
| RR, breaths/min, median (IQR) | 28 (24-35) | 30 (24-37) | 0.602 |  |
| SaO_2_, %, median (IQR) | 88 (80-91) | 86 (82-90) | 0.950 |  |
| Fever ≥38^o^ C (≥100.4^o^ F), n (%) | 27 (54) | 16 (48) | 0.645 |  |
| qSOFA, n (%) |  |  |  |  |
| 0 | 3 (6) | 0 (0) | 0.273 |  |
| 1 | 34 (68) | 26 (79) | 0.283 |  |
| 2 | 7 (14) | 6 (18) | 0.608 |  |
| 3 | 6 (12) | 1 (3) | 0.235 |  |
| Lung injury on chest CT scan, %, median (IQR) | 65 (51-76) | 62 (52-76) | 0.821 |  |
| CRP, mg/L, median (IQR) | 151 (103-228) | 173 (94-260) | 0.428 |  |
| Ferritin, ng/mL, median (IQR) | 691 (604-720) | 687 (434-714) | 0.673 |  |
| Interleukin-6, pg/mL, median (IQR) | 80 (35-142) | 109 (33-242) | 0.369 |  |
| Procalcitonin ≥0.5 ng/mL, n (%) | 12 / 45 (27) | 11 / 29 (38) | 0.307 |  |
| d-dimer, ng/mL, median (IQR) | 1,175 (597-2,256) | 2,069 (1,042-3,912) | **0.048** |  |
| Creatinine, mcmol/L, median (IQR) | 87 (75-107) | 103 (77-129) | 0.123 |  |
| Arterial blood gas analysis, median (IQR) |  |  |  |  |
| Blood pH | 7.42 (7.35-7.46) | 7.38 (7.20-7.46) | 0.158 |  |
| PaO_2_, mm Hg | 75 (67-101) | 88 (78-112) | 0.220 |  |
| PaCO_2_, mm Hg | 39 (35-43) | 41 (29-54) | 0.783 |  |
| Complete blood count, median (IQR) |  |  |  |  |
| Hemoglobin, g/L | 129 (114-138) | 123 (107-136) | 0.339 |  |
| Leukocytes x10^9^/L | 7.9 (6.0-10.9) | 8.6 (6.0-13.1) | 0.308 |  |
| Neutrophils x10^9^/L | 6.4 (4.6-8.8) | 7.5 (5.2-10.9) | 0.308 |  |
| Lymphocytes x10^9^/L | 0.7 (0.5-1.1) | 0.6 (0.3-1.0) | 0.095 |  |
| Platelets x10^9^/L | 230 (167-285) | 233 (175-328) | 0.266 |  |
| BMI, body mass index; BP, blood pressure; CRP, C-reactive protein; CT, computed tomography; CVD, cardiovascular disease; HR, heart rate; ICU, intensive care unit; IQR, interquartile range; PaCO2, partial pressure of carbon dioxide; PaO2, partial pressure of oxygen; qSOFA, quick sequential organ failure assessment score; RR, respiratory rate; RT-PCR, reverse transcription polymerase chain reaction; SaO2, oxygen saturation of arterial blood; SARS-CoV-2, severe acute respiratory syndrome coronavirus 2 | | | | |

**TABLE S6.** Management and outcomes of a dexamethasone cohort

| **Variable** | | | **<65 years**  (n=50) | | **≥65 years**  (n=33) | | ***p* value** | | |
| --- | --- | --- | --- | --- | --- | --- | --- | --- | --- |
| Dexamethasone therapy | |  |  | |  | |  | | |
| Therapy start*****, days, median (IQR) | | | 10 (8-13) | | 11 (8-15) | | 0.565 | | |
| Duration of therapy, days, median (IQR) | | | 3 (3-3) | | 3 (3-4) | | 0.104 | | |
| Cumulative dose, mg, median (IQR) | | | 36 (24-36) | | 36 (36-48) | | 0.190 | | |
| Hydroxychloroquine, n (%) | | | 19 (32) | | 11 (14) | | **0.015** | | |
| Sarilumab, n (%) | | | 3 (5) | | 5 (6) | | 1.000 | | |
| Tocilizumab, n (%) | | | 10 (17) | | 3 (4) | | **0.011** | | |
| Antibiotics, n (%) | | | 49 (98) | | 32 (97) | | 1.000 | | |
| DVT prophylaxis, n (%) | | | 50 (100) | | 33 (100) | | NA | | |
| RBC transfusion, n (%) | | 11 (22) | | 10 (20) | | 0.394 | | |  |
| Dialysis, n (%) | | 13 (26) | | 11 (33) | | 0.471 | | |  |
| Vasopressor agents, n (%) | | | 27 (54) | | 27 (82) | | **0.009** | | |
| NEpi only, n (%) | | | 20 (40) | | 14 (42) | | 0.112 | | |
| NEpi + Epi, n (%) | | | 6 (12) | | 12 (36) | |  |  |  |
| NEpi + Epi + other agents, n (%) | | | 1 (2) | | 1 (3) | |  |  |  |
| Highest dose, mcg/kg, median (IQR) | | | 0.4 (0.2-1.2) | | 0.8 (0.6-1.0) | | 0.112 | | |
| Nutrition, n (%) | | |  | |  | |  | | |
| Oral intake | | | 15 (30) | | 5 (15) | | 0.122 | | |
| NG feeding tube | | | 24 (48) | | 26 (79) | | **0.005** | | |
| NG feeding tube + PN | | | 11 (22) | | 2 (6) | | 0.051 | | |
| O_2_ therapy, n (%) | |  | |  | |  | | |  |
| Nasal cannula | | 33 (66) | | 21 (64) | | 0.825 | | |  |
| HFNC | | 10 (20) | | 5 (15) | | 0.574 | | |  |
| Non-invasive mechanical ventilation | | 10 (20) | | 8 (24) | | 0.646 | | |  |
| Intubation, n (%) | | 31 (62) | | 28 (85) | | **0.025** | | |  |
| Tracheostomy, n (%) | | 30 / 31 (97) | | 16 / 28 (57) | | 0.302 | | |  |
| Day of tracheostomy after intubation, n (%) | | 6 (4-7) | | 5 (3-7) | | 0.296 | | |  |
| ECMO, n (%) | | | 1 (2) | | 0 (0) | | 1.000 | | |
| Extubation / Decannulation, n (%) | | 21 / 31 (68) | | 6 / 28 (21) | | **<0.001** | | |  |
| Duration of ventilation, days, median (IQR) | | 20 (12-33) | | 11 (8-18) | | **0.002** | | |  |
| Bleeding, n (%) | | 13 (26) | | 9 (27) | | 0.898 | | |  |
| Non-ventricular arrhythmia, n (%) | | 7 (14) | | 19 (58) | | **<0.001** | | |  |
| Ventricular arrhythmia, n (%) | | 1 (2) | | 0 (0) | | 1.000 | | |  |
| *Clostridioides difficile* infection, n (%) | | 3 (6) | | 2 (6) | | 1.000 | | |  |
| Thromboembolic events, n (%) | | 12 (24) | | 5 (15) | | 0.328 | | |  |
| Hyperglycemia, n (%) | | 13 (26) | | 20 (61) | | **0.002** | | |  |
| Bacterial infection, n (%) | | 31 (62) | | 28 (85) | | **0.025** | | |  |
| Septic shock, n (%) | | 14 (28) | | 25 (76) | | **<0.001** | | |  |
| Initial ICU stay, days, median (IQR) | | 12 (3-24) | | 10 (4-18) | | 0.762 | | |  |
| ICU readmission, n (%) | | 2 (4) | | 3 (9) | | 0.387 | | |  |
| Total ICU stay, days, median (IQR) | | 12 (3-24) | | 10 (5-18) | | 0.863 | | |  |
| Length of hospital stay, days, median (IQR) | | 22 (15-38) | | 15 (11-29) | | **0.035** | | |  |
| 28-day ICU mortality, n (%) | | 7 (14.0) | | 21 (63.6) | | **<0.001** | | |  |
| Outcome, n (%) | |  | |  | |  | | |  |
| Discharged | | 40 (80.0) | | 11 (33.3) | | **<0.001** | | |  |
| Deceased | | 10 (20.0) | | 22 (66.7) | |  |  |  |  |
| DVT, deep vein thrombosis; ECMO, extracorporeal membrane oxygenation; Epi, epinephrine; HFNC, high-flow nasal cannula; ICU, intensive care unit; IQR, interquartile range; NA, not applicable; NEpi, norepinephrine; NG, nasogastric; PN, parenteral nutrition; RBC, red blood cells  *** since the first day of fever** | | | | | | | | |  |

**TABLE S7.** Characteristics of a usual care cohort at ICU admission

| **Variable** | **<65 years**  (n=60) | **≥65 years**  (n=77) | ***p* value** |  |
| --- | --- | --- | --- | --- |
| Age, years, median (IQR) | 54 (48-61) | 74 (70-82) | **<0.001** |  |
| Female sex, n (%) | 21 (35) | 36 (47) | 0.166 |  |
| BMI ≥30, kg/m2, n (%) | 29 / 41 (71) | 23 / 51 (45) | **0.014** |  |
| Positive RT-PCR SARS-CoV-2, n (%) | 33 (55) | 50 (65) | 0.238 |  |
| Comorbidities, n (%) | 39 (65) | 72 (94) | **<0.001** |  |
| Hypertension | 28 (47) | 60 (78) | **<0.001** |  |
| Other CVD | 12 (20) | 34 (44) | **0.003** |  |
| Chronic lung disease | 6 (10) | 10 (13) | 0.585 |  |
| Chronic kidney disease | 2 (3) | 9 (12) | 0.111 |  |
| Diabetes mellitus | 15 (25) | 26 (34) | 0.257 |  |
| Malignancy | 5 (8) | 10 (13) | 0.398 |  |
| Systolic BP, mm Hg, median (IQR) | 130 (118-140) | 130 (115-140) | 0.540 |  |
| Diastolic BP, mm Hg, median (IQR) | 75 (69-80) | 75 (65-82) | 0.566 |  |
| HR, beats/min, median (IQR) | 86 (75-100) | 82 (74-98) | 0.262 |  |
| RR, breaths/min, median (IQR) | 30 (23-35) | 24 (20-28) | **0.005** |  |
| SaO_2_, %, median (IQR) | 88 (81-94) | 90 (84-94) | 0.288 |  |
| Fever ≥38^o^ C (≥100.4^o^ F), n (%) | 26 (43) | 25 (32) | 0.286 |  |
| qSOFA, n (%) |  |  |  |  |
| 0 | 8 (13) | 8 (10) | 0.595 |  |
| 1 | 35 (58) | 38 (49) | 0.296 |  |
| 2 | 9 (15) | 20 (26) | 0.119 |  |
| 3 | 8 (13) | 11 (14) | 0.873 |  |
| Lung injury on chest CT scan, %, median (IQR) | 59 (42-73) | 52 (32-75) | 0.177 |  |
| CRP, mg/L, median (IQR) | 121 (66-215) | 115 (53-201) | 0.531 |  |
| Ferritin, ng/mL, median (IQR) | 658 (505-711) | 575 (361-697) | 0.234 |  |
| Interleukin-6, pg/mL, median (IQR) | 73 (17-292) | 95 (44-212) | 0.507 |  |
| Procalcitonin ≥0.5 ng/mL, n (%) | 17 / 48 (35) | 18 / 62 (29) | 0.476 |  |
| d-dimer, ng/mL, median (IQR) | 1,418 (586-3,325) | 1,738 (1,012-4,300) | 0.138 |  |
| Creatinine, mcmol/L, median (IQR) | 83 (69-105) | 97 (74-136) | 0.060 |  |
| Arterial blood gas analysis, median (IQR) |  |  |  |  |
| Blood pH | 7.39 (7.31-7.43) | 7.38 (7.32-7.44) | 0.944 |  |
| PaO_2_, mm Hg | 89 (60-99) | 88 (70-114) | 0.286 |  |
| PaCO_2_, mm Hg | 40 (32-53) | 38 (32-50) | 0.712 |  |
| Complete blood count, median (IQR) |  |  |  |  |
| Hemoglobin, g/L | 134 (118-144) | 118 (90-136) | **0.012** |  |
| Leukocytes x10^9^/L | 8.6 (4.6-12.9) | 8.0 (5.5-11.8) | 0.945 |  |
| Neutrophils x10^9^/L | 6.7 (3.3-10.4) | 7.2 (4.3-10.4) | 0.638 |  |
| Lymphocytes x10^9^/L | 0.8 (0.5-1.3) | 0.7 (0.4-1.0) | 0.251 |  |
| Platelets x10^9^/L | 209 (169-283) | 189 (137-279) | 0.393 |  |
| BMI, body mass index; BP, blood pressure; CRP, C-reactive protein; CT, computed tomography; CVD, cardiovascular disease; HR, heart rate; ICU, intensive care unit; IQR, interquartile range; PaCO2, partial pressure of carbon dioxide; PaO2, partial pressure of oxygen; qSOFA, quick sequential organ failure assessment score; RR, respiratory rate; RT-PCR, reverse transcription polymerase chain reaction; SaO2, oxygen saturation of arterial blood; SARS-CoV-2, severe acute respiratory syndrome coronavirus 2 | | | | |

**TABLE S8.** Management and outcomes of a usual care cohort

| **Variable** | | | **<65 years**  (n=60) | | **≥65 years**  (n=77) | | ***p* value** | | |
| --- | --- | --- | --- | --- | --- | --- | --- | --- | --- |
| Hydroxychloroquine, n (%) | | | 19 (32) | | 11 (14) | | **0.015** | | |
| Sarilumab, n (%) | | | 3 (5) | | 5 (6) | | 1.000 | | |
| Tocilizumab, n (%) | | | 10 (17) | | 3 (4) | | **0.011** | | |
| Antibiotics, n (%) | | | 59 (98) | | 74 (96) | | 0.631 | | |
| DVT prophylaxis, n (%) | | | 57 (95) | | 75 (97) | | 0.653 | | |
| RBC transfusion, n (%) | | 12 (20) | | 15 (19) | | 0.940 | | |  |
| Dialysis, n (%) | | 11 (18) | | 16 (21) | | 0.721 | | |  |
| Vasopressor agents, n (%) | | | 27 (45) | | 39 (51) | | 0.511 | | |
| NEpi only, n (%) | | | 18 (30) | | 23 (30) | | 0.814 | | |
| NEpi + Epi, n (%) | | | 8 (13) | | 14 (18) | |  |  |  |
| NEpi + Epi + other agents, n (%) | | | 1 (2) | | 2 (3) | |  |  |  |
| Highest dose, mcg/kg, median (IQR) | | | 0.6 (0.2-1.0) | | 0.6 (0.2-1.0) | | 0.816 | | |
| Nutrition, n (%) | | |  | |  | |  | | |
| Oral intake | | | 27 (45) | | 33 (43) | | 0.802 | | |
| NG feeding tube | | | 33 (55) | | 38 (49) | | 0.511 | | |
| NG feeding tube + PN | | | 0 (0) | | 6 (8) | | **0.035** | | |
| O_2_ therapy, n (%) | |  | |  | |  | | |  |
| Nasal cannula | | 44 (57) | | 58 (75) | | 0.791 | | |  |
| HFNC | | 11 (14) | | 15 (19) | | 0.865 | | |  |
| Non-invasive mechanical ventilation | | 2 (3) | | 3 (4) | | 1.000 | | |  |
| Intubation, n (%) | | 33 (55) | | 38 (49) | | 0.511 | | |  |
| Tracheostomy, n (%) | | 23 / 33 (70) | | 17 / 38 (45) | | **0.038** | | |  |
| Day of tracheostomy after intubation, n (%) | | 5 (3-10) | | 7 (3-19) | | 0.533 | | |  |
| ECMO, n (%) | | | 2 (3) | | 0 (0) | | 0.183 | | |
| Extubation / Decannulation, n (%) | | 14 / 33 (42) | | 9 / 38 (24) | | 0.092 | | |  |
| Duration of ventilation, days, median (IQR) | | 12 (5-17) | | 13 (4-21) | | 0.665 | | |  |
| Bleeding, n (%) | | 9 (15) | | 7 (9) | | 0.285 | | |  |
| Non-ventricular arrhythmia, n (%) | | 9 (15) | | 17 (22) | | 0.295 | | |  |
| Ventricular arrhythmia, n (%) | | 0 (0) | | 4 (5) | | 0.131 | | |  |
| *Clostridioides difficile* infection, n (%) | | 1 (2) | | 4 (5) | | 0.385 | | |  |
| Thromboembolic events, n (%) | | 11 (18) | | 13 (17) | | 0.825 | | |  |
| Hyperglycemia, n (%) | | 14 (23) | | 18 (23) | | 0.995 | | |  |
| Bacterial infection, n (%) | | 39 (65) | | 45 (58) | | 0.434 | | |  |
| Septic shock, n (%) | | 16 (27) | | 28 (36) | | 0.228 | | |  |
| Initial ICU stay, days, median (IQR) | | 6 (2-16) | | 4 (2-13) | | 0.606 | | |  |
| ICU readmission, n (%) | | 3 (5) | | 9 (12) | | 0.169 | | |  |
| Total ICU stay, days, median (IQR) | | 6 (2-16) | | 5 (2-14) | | 0.872 | | |  |
| Length of hospital stay, days, median (IQR) | | 19 (12-26) | | 17 (12-27) | | 0.773 | | |  |
| 28-day ICU mortality, n (%) | | 16 (26.7) | | 38 (49.4) | | **0.007** | | |  |
| Outcome, n (%) | |  | |  | |  | | |  |
| Discharged | | 41 (68.3) | | 37 (48.1) | | **<0.001** | | |  |
| Deceased | | 19 (31.7) | | 40 (51.9) | |  |  |  |  |
| DVT, deep vein thrombosis; ECMO, extracorporeal membrane oxygenation; Epi, epinephrine; HFNC, high-flow nasal cannula; ICU, intensive care unit; IQR, interquartile range; NA, not applicable; NEpi, norepinephrine; NG, nasogastric; PN, parenteral nutrition; RBC, red blood cells | | | | | | | |  |  |
